# Negative rumination in depression subtypes with melancholic features and anxious distress

**DOI:** 10.3389/fpsyg.2025.1515500

**Published:** 2025-02-13

**Authors:** Hong-li Wang, Xiao-ning Shi, Jin-long Zhao, Qiong Jia, Wei Xu, Wen-wen Dun, Ying-ying Zhao

**Affiliations:** The National Clinical Research Center for Mental Disorders and Beijing Key Laboratory of Mental Disorders, Beijing Anding Hospital, Advanced Innovation Center for Human Brain Protection and Laboratory for Clinical Medicine, Capital Medical University, Beijing, China

**Keywords:** major depression, negative cognitive bias, subtype, melancholic, anxious distress

## Abstract

**Background:**

Aberrant cognition is one of the crucial symptoms of depression. However, whether the negative rumination participates in depression with melancholic features or anxious distress remains unclear.

**Methods:**

In this study, we addressed this issue by compiling a questionnaire that can comprehensively measure the negative cognitive processing bias in depression. We also conducted an exhaustive analysis of its influencing factors, including the subtype of depression, age, gender, age of onset, family history of mental disorder, and education year.

**Results:**

We found that depression increased negative attention bias, negative memory bias, negative interpretation bias, and negative rumination bias. Importantly, among the several dimensions of negative cognitive bias, negative rumination bias was more serious in the melancholic than anxious subgroup. Furthermore, Spearman rank correlation analysis showed that negative rumination bias correlates with family history and age of onset of depression.

**Limitations:**

We mainly explored melancholic and anxiety subgroups and did not include other subtypes. Due to time constraints, we did not conduct long-term follow-ups or explore the neural mechanisms of the differences between depressive and anxious rumination.

**Conclusion:**

Our results contribute to the existing literature on the psychological mechanisms underlying aberrant cognition in depression. These findings could provide guidance for clinical practice and individualized precision treatment of cognitive biases in major depressive disorder. Therefore, rumination-focused therapies would be tailored differently for melancholic versus anxious subgroups.

## 1 Introduction

Major depressive disorder (MDD) affects almost 350 million people globally, causing disability and a tremendous burden on families and society (Huang et al., 2019; Smith, 2014). However, MDD is a highly heterogeneous disorder (Uher et al., 2014; Zimmerman et al., 2015), with its pathophysiology remaining unclear. According to the *Diagnostic and Statistical Manual of Mental Disorders, 5th edition (DSM-5)*, depression is divided into several subtypes. The specifier “with melancholic features” is characterized by profound hopelessness, despair, moroseness, or empty mood. Another specifier, “with anxious distress,” is defined as the presence of two or more of five anxious symptoms during depression (Uher et al., 2014). Recently, there has been increasing recognition of cognitive dysfunction as a core feature of depression. Patients with MDD having higher polygenic risk scores were more likely to exhibit melancholic features (Oliva et al., 2023). Melancholic patients were more severely depressed and characterized by worse cognitive performance in attention/working memory, visual learning, reasoning/problem-solving (Zaninotto et al., 2016), and information speed, decision speed, and reward-relevant emotional processing of happy expressions, even after co-varying for symptom severity. Anxious distress is known to have different neurobiological profiles compared to non-anxious depression regarding the hypothalamic–pituitary–adrenal axis function, structural and functional brain imaging findings, inflammation markers, and so on (Choi et al., 2020).

In MDD, aberrant cognition strongly contributes to functional impairments but is barely addressed in current therapies (Monroe and Harkness, 2022). The cognitive dysfunction is usually persistent and recurrent and significantly reduces the quality of life in patients with depression (Chamberlain and Sahakian, 2004). Indeed, negative cognitive biases may contribute to the maintenance of specific depression symptoms, such as sadness, hopelessness, guilt, pessimism, and indecision (Beevers et al., 2019). Beck’s cognitive bias theory of depression is that adverse life events result in negative automatic thinking; that is, MDD usually has cognitive distortion in perception, attention, memory, and reasoning (Kovacs and Beck, 1978; Pössel and Black, 2014). These negative biases are one of the core features of depression, including pessimistic views on self, environment, and future (Phillips et al., 2003). Indeed, depressed patients are more likely to recall negative autobiographical memories and rarely think of delighted times (Brittlebank et al., 1993). Korn et al. reported a lack of optimistic belief in depressed individuals, and this absence correlated with symptom severity (Korn et al., 2014). Depressive symptoms were positively and strongly correlated with negative automatic thoughts about self and moderately associated with dysfunctional attitudes among people living on the four continents.

The cognitive model of depression, including biased attention, rumination, memory, and dysfunctional schemas, has been strongly and consistently related to its onset and maintenance (Disner et al., 2011). In addition, rumination characterized by repetitive negative thinking is one of the risk factors for MDD. These negative thoughts usually have an intimate association with adverse outcomes in depression, including its onset, severity, and therapeutic efficacy (Wahl et al., 2019). Park and his colleagues demonstrated the association between higher rumination intensity and greater neural activity in frontoparietal regions responsible for cognitive control (Park et al., 2024). Moreover, the study reported the link between rumination and default mode network (DMN) activation, especially in the DMN core regions and the dorsal medial prefrontal cortex (Zhou et al., 2020). Since rumination is intertwined with the difficulty of regulating negative thoughts in MDD, the association between rumination and its subtypes has not been studied. The two most important characteristics of negatively biased cognition include pessimistic judgment bias (Douglas and Porter, 2010; Maniglio et al., 2014) and catastrophic reactions to negative feedback (Beats et al., 1996; Elliott et al., 1997). There is a study to examine the role of a negative interpretation bias in adolescent pain (Heathcote et al., 2016). In the cross-modal, the selective attentional bias occurs both in the engagement and the disengagement facets (Mao et al., 2020). Among the different subtypes of depression, patients with melancholic and anxious distress characteristics had higher morbidity and suicide risk than other subtypes, and negative bias was a prominent and common stress-coping pattern in the two subtypes (Smith et al., 2018; Sokol et al., 2022; Vinograd et al., 2020). Negative bias is one of the important psychopathological features of depression (Dai et al., 2019; Winer and Salem, 2016), which manifests differently in different subtypes. Previous studies have found that patients with anxious distress have significant negative attention and interpretation biases (Elgersma et al., 2018), and patients with melancholic traits have more significant negative memory and rumination biases (Marchetti et al., 2018). Therefore, negative memory and rumination bias are strongly associated with melancholic characteristics, and negative attention bias is closely related to anxious distress, but more evidence is needed. The negative cognitive processing bias questionnaire is used for the early warning and diagnosis of depression, and it could evaluate the extent of cognitive impairment in depressive patients.

However, whether the rumination is involved in the negative cognition bias in depression is not clear. Therefore, it is vital to comprehensively measure the negative cognitive processing bias in depression and analyze its influencing factors, including the subtypes of MDD, age, gender, age of onset, family history of mental disorder, education year, and so on.

## 2 Methods and materials

### 2.1 Participants and study setting

This is an observational, cross-sectional study. The inpatients aged 15–65 were recruited from 5 wards of the depression treatment center in Beijing Anding Hospital. Patients were enrolled using the continuous sampling method, as reported (Shi et al., 2023). While the continuous sampling method is common in observational studies, it may introduce selection bias. Inclusion and exclusion criteria were strictly followed to minimize selection bias. The patients diagnosed with major depressive disorder and who meet the subtype criteria of “with melancholic features” or “with anxious distress” according to DSM-5 were included. Patients with comorbid obsessive-compulsive disorder or substance abuse, or those unable to complete the questionnaire, were excluded. Besides, another group of subjects aged 15–65, with no previous history of mental illness, was recruited as a control group.

### 2.2 Assessment procedure

The depressed inpatients were required to complete the negative cognition processing bias questionnaire within 3 days after hospitalization. The people in the control group were also asked to complete the same questionnaire. The negative cognition processing bias questionnaire that fits Chinese culture was used in this program. It was formed after item analysis and factor analysis and has been tested nationwide in China. It has been proven reliable and valid (Miao et al., 2022). Cronbach’s alpha and McDonald’s omega showed good internal consistency reliability for this questionnaire, including the whole scale and its subdimensions (both α and ω = 0.866). Regarding its validity, the scores for both DAS (*r* = 0.551, *p* < 0.001) and BDI-II (*r* = 0.447, *p* < 0.001) were significantly correlated with the total score of the questionnaire and even its subdimensions. The four components of negative cognitive processing bias are negative attention bias, negative memory bias, negative interpretation bias, and negative rumination bias (Beal et al., 2023).

The demographic information, medical history, and disease characteristics of the MDD patients were recorded. The selected variables are as follows: subtypes of depression (with melancholic features or with anxious distress), gender, age, education year, age of onset, occupational status (employed/part-time/unemployed/student), family history of mental disorder, first episode or recurrence, and with or without psychotic symptoms. The study protocol was approved by the independent ethics committee of Beijing Anding Hospital.

### 2.3 Statistical analysis

The counting data were presented as means ± standard deviations (SD). The total score and the four different dimension score of the negative cognition processing bias were calculated. Homogeneity of variance and whether the data conform to normal distribution were tested. Gender differences were compared using a chi-squared test. An independent-samples *t*-test was used to analyze differences between the control and MDD groups, as well as the differences between the two subtypes with melancholic features or with anxious distress of MDD. Correlation analysis was used to explore the factors influencing the negative cognition processing bias. Non-parametric tests were performed on data that did not conform to normal distribution. Spearman’s rank correlation analysis was employed to identify the correlation between negative cognitive bias and age, age of onset, and years of education. The analysis was conducted using SPSS Statistics 26, with the level of significance set at 0.05, which is a two-tailed analysis.

## 3 Results

### 3.1 Demographic and clinical characteristics of the sample

In total, 154 subjects were included in the study. A total of 112 patients who fulfilled the inclusion criteria were recruited as the MDD group, with 56 depressed patients fitting the “with the melancholic feature” (Mel subgroup) and 56 fitting the “with anxious distress feature” (Anx subgroup). Additionally, 42 healthy subjects were recruited as a control group. The demographic and clinical characteristics of the subjects are presented in Table 1. The chi-squared test revealed that the sex distribution of MDD and the control group was not statistically significant (χ^2^ = 0.088, *p* = 0.767). The Mann–Whitney U test showed no significant statistical difference in age (*Z* = −0.708, *p* = 0.479) and education year (*Z* = 1.793, *p* = 0.073) between the MDD and control groups. The chi-squared test revealed that sex distribution (χ^2^ = 0.322, *p* = 0.570), recurrence (χ^2^ = 0.144, *p* = 0.705), family history (χ^2^ = 0.156, *p* = 0.693), and psychotic symptoms (χ^2^ = 0.019, *p* = 0.891) of the melancholic and anxious groups were not statistically significant. The Mann–Whitney U test showed no significant statistical difference in age (*Z* = 0.346, *p* = 0.729), age of onset (*Z* = −0.047, *p* = 0.963), time of onset (*Z* = 0.640, *p* = 0.522), and education year (*Z* = −0.819, *p* = 0.413) between the melancholic and anxious groups.

**Table 1:** Demographic and clinical characteristics of MDD, with melancholic features (Melancholic), with anxious distress (Anxious), and the control groups (Control).

Variable	MDD			Control
	Total	Melancholic	Anxious	
Number of subjects	112	56	56	42
Age	35.75 ± 15.85	35.16 ± 16.36	36.34 ± 15.38	32.26 ± 10.54
Female (%)	53 (47.32)	25 (22.32)	28 (25.00)	21 (50)
Years of education	13.72 ± 3.58	13.98 ± 3.53	13.46 ± 3.63	14.98 ± 3.96
Age of first onset	29.30 ± 14.34	29.16 ± 14.19	29.45 ± 14.63	–
Time of onset	2.68 ± 2.59	2.70 ± 2.97	2.67 ± 2.16	–
Recurrence (%)	52 (46.43)	27 (24.11)	25 (22.32)	–
Family history (%)	40 (35.71)	19 (16.96)	21 (18.75)	–
With psychotic symptoms (%)	23 (20.5)	11 (9.82)	12 (10.71)	–
**Occupation**				
Employed (%)	52 (46.42)	24 (21.43)	28 (25.00)	26 (61.90)
Part-time (%)	2 (1.79)	1 (0.89)	1 (0.89)	2 (4.76)
Student (%)	35 (31.25)	21 (18.75)	14 (12.50)	12 (28.57)
Retired (%)	8 (7.14)	5 (4.46)	3 (2.68)	2 (4.76)
Unemployed (%)	15 (13.39)	5 (4.46)	10 (8.93)	0 (0)

### 3.2 Negative cognitive processing bias of the subjects

**Figure 1:**
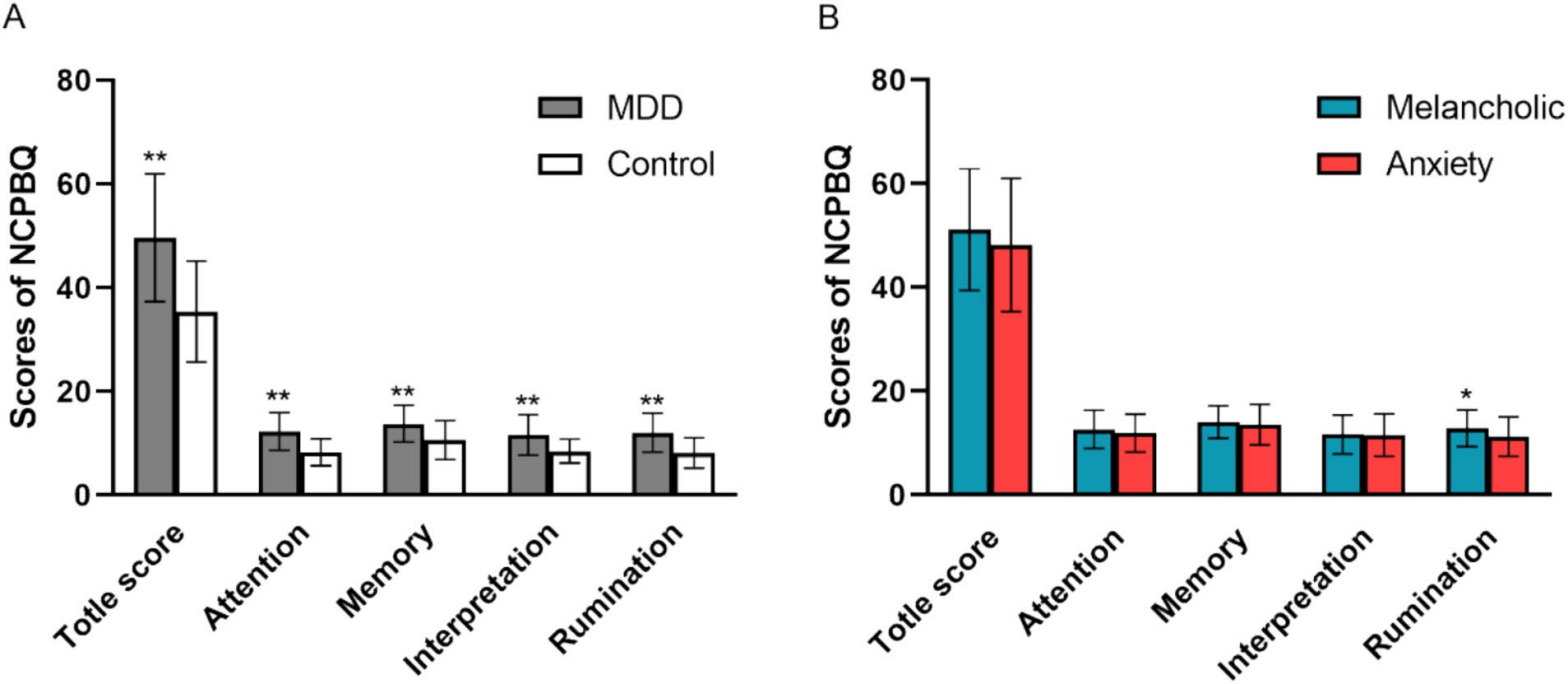
Total score and the subjects’ four components of negative cognitive processing bias. **(A)** Negative cognitive processing bias between MDD and Control. **(B)** Negative cognitive processing bias between two MDD subgroups of “with a melancholic feature” and “with anxious distress.” NCPBQ, negative cognition processing bias questionnaire; MDD, major depressive disorder. *p < 0.05, **p < 0.01.

The total score and the scores of the four components of negative cognitive processing bias of the subjects are shown in Figure 1.

#### 3.2.1 Difference of negative cognitive processing bias between MDD and control groups

The Kolmogorov–Smirnov one-sample test (K-S normality test) showed that the total negative bias score, negative attention, negative memory, and negative rumination dimension data of the control group (*n* = 42) did not conform to normal distribution. The dimensions of negative memory, negative interpretation, and negative rumination in the depression group (*n* = 112) did not conform to the normal distribution. Therefore, non-parametric tests were performed to compare the differences between the two groups. The results showed that there were significant statistical differences in the total score of negative bias (*Z* = −6.038, *p* < 0.000) and each dimension of negative bias, such as negative attention (*Z* = −5.989, *p* < 0.000), negative interpretation (*Z* = −4.600, *p* < 0.000), negative memory (*Z* = −4.252, *p* < 0.000), and negative rumination (*Z* = −5.740, *p* < 0.000) between the depression group and the control group (independent sample Mann–Whitney U test) (Figure 1A).

#### 3.2.2 Difference of negative cognitive processing bias between two MDD subgroups of “with the melancholic feature” and “with anxious distress”

The K-S one sample test showed that negative attentional bias and negative interpretation in the melancholic group did not conform to the normal distribution; therefore, the non-parametric test was performed to compare the differences between the two subgroups. Independent samples *t*-test showed a statistically significant difference in negative rumination bias between the melancholic and anxiety subgroups (*t* = 2.33, *p* = 0.022) (Figure 1B). The independent sample Mann–Whitney U test showed that there was no significant difference in negative interpretation and negative attention bias between the melancholic subgroup and the anxiety subgroup. Similarly, the independent samples *t-*test showed no significant difference in the total score and its component negative memory bias.

### 3.3 Clinical characteristics associated with the negative cognitive processing bias in patients with MDD

**Figure 2:**
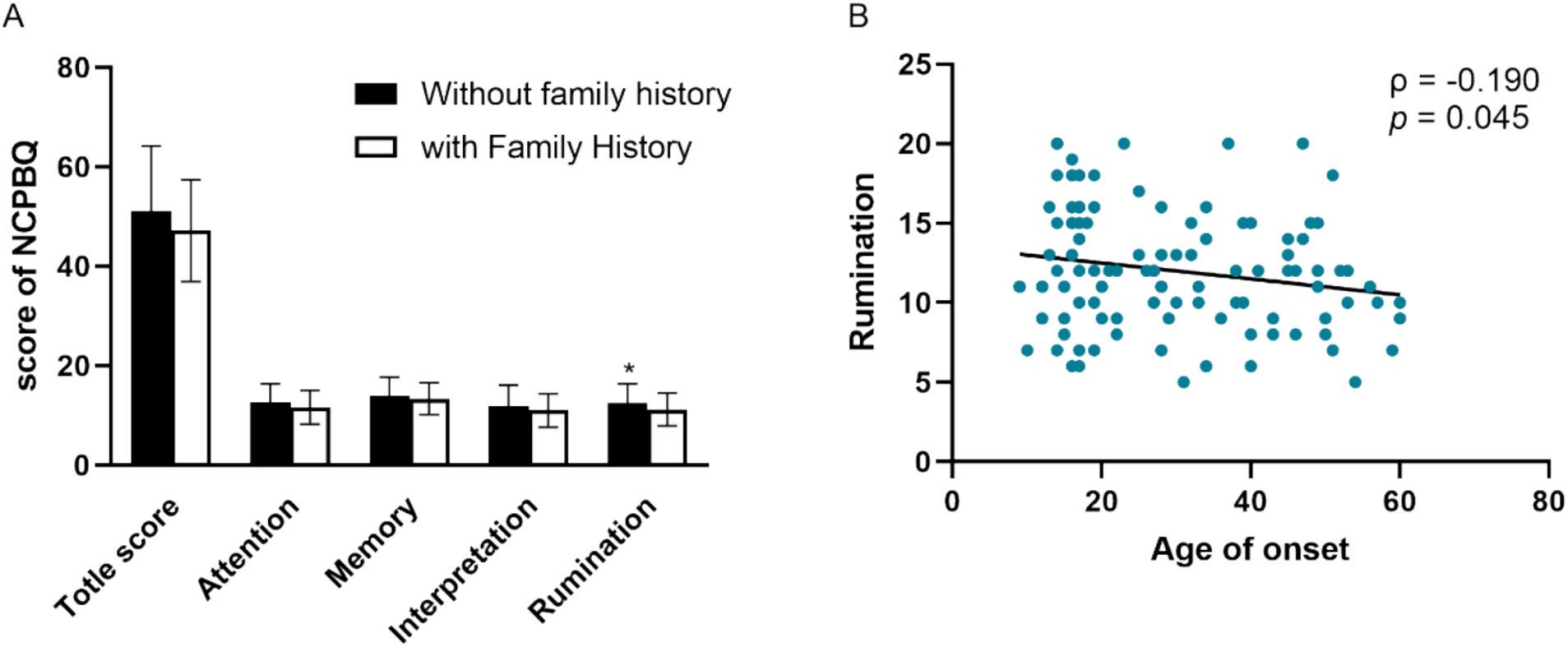
Clinical characters associated with the negative cognitive processing bias in patients with MDD. **(A)** Negative rumination bias correlates with family history. **(B)** Negative rumination bias correlates with age of onset. NCPBQ: negative cognition processing bias questionnaire. MDD: major depressive disorder. *p < 0.05.

The Kolmogorov–Smirnov test showed that the data of age, age of onset, and education year did not conform to normal distribution. Spearman’s rank correlation analysis showed that negative rumination bias correlates with family history (*ρ* = −0.187, *p* = 0.049) and age of onset (ρ = −0.190, *p* = 0.045) (Figure 2). Moreover, age was correlated with the total score of negative bias (ρ = −0.245, *p* = 0.009), negative interpretation (ρ = −0.196, *p* = 0.038), and negative memory bias (ρ = −0.286, *p* = 0.002). Age of onset correlates with a total score of negative bias (ρ = −0.271, *p* = 0.004), negative memory bias (ρ = −0.286, *p* = 0.002), and negative interpretation (ρ = −0.229, *p* = 0.015). The educated year correlates with negative memory bias (ρ = −0.240, *p* = 0.011). There were no significant differences in negative bias among patients with the first episode or recurrence, as well as patients with or without psychotic symptoms.

## 4 Discussion

In the present study, we investigated the involvement of negative cognitive processing bias in major depressive disorder. We found that depression increased the total scores and each dimension of negative cognitive processing bias. Importantly, among the several dimensions of negative cognitive bias, rumination in the melancholic subgroup was more serious than in the anxiety subgroup. Furthermore, negative rumination bias correlates with family history and age of onset. Researchers suggest that decline in rumination may be a common feature following cognitive behavior therapy interventions. Moreover, rumination scores were associated with post-treatment mindfulness-based cognitive therapy. These findings could provide guidance for clinical practice and contribute to the understanding of cognitive biases in major depressive disorder. Therefore, rumination-focused therapies could be tailored differently for melancholic versus anxious subgroups. Our research provides evidence for individualized precision treatment.

Our finding that depression increased the negative cognitive processing bias is consistent with the results of a previous study (Li et al., 2022). It might be argued that healthy people deal with life events with a more positive attitude. However, negative processing bias is a cognitive trait that depressed patients prefer negative information. Similarly, the four components of negative cognitive processing bias, including negative attention bias, negative memory bias, negative interpretation bias, and negative rumination bias, were more serious than controls. Depression has two domains of biased cognition: pessimistic judgment bias and aberrant response to negative feedback (Noworyta et al., 2021). Abnormal neural processing and negative emotional bias are trait marks of depression (Li et al., 2022). Negative bias may be facilitated by the excessive ventral bottom-up negative emotion along with the incapability of the dorsal prefrontal top-down system (Noworyta et al., 2021).

Rumination is one of the crucial features of depression (Zhang et al., 2020). So far, patients are often treated homogeneously despite having heterogeneous symptoms with distinct underlying neural mechanisms. Treatment that is directly relevant to an individual patient’s subset of symptoms might more precisely and thus effectively aid in the alleviation of their specific symptoms. Our results showed that negative rumination bias in the melancholic subgroup is more serious than that in the anxiety subgroup. These results are consistent with previous studies. Nelson et al. proposed that ruminative thinking could distinguish melancholic from non-melancholic major depression. They found that ruminative thinking was present in 53% of patients with melancholia but only 11% of the non-melancholic patients. Rumination appears to be useful for making the diagnosis of melancholia and may facilitate the study of the psychobiology and treatment of this disorder (Nelson and Mazure, 1985). Patients who engage in rumination may focus more on adverse life events, gloomy emotions, and the possible terrible consequences and could not distract themselves from disruptive moods promptly. Then, rumination exacerbates and prolongs depressed symptoms in vulnerable individuals via the following mechanisms (Nolen-Hoeksema et al., 2008). Firstly, rumination intensifies pessimistic thoughts and biological responses to stress. Secondly, depression with a ruminative tendency leads to negative thinking and difficulty in solving problems effectively. Lastly, ruminative patients obtain less social support, which further increases depressive symptoms (Watkins, 2008), depressive episodes (Nolen-Hoeksema et al., 2008), and risk of relapse (Nolen-Hoeksema, 2000). Removing negative bias from patients with depression can modulate the abnormal physiological stress responses (Jopling et al., 2020). Depressed patients have more biological factors that cause more negative rumination. Moreover, Brooke et al. regard rumination as a mediator of metacognitive training for depression in older adults (Schneider et al., 2023). Mindfulness-based cognitive therapy led to decreased salience network connectivity to the lingual gyrus during a ruminative state, and this change mediated improvements in the ability to sustain and control attention to body sensations (van der Velden et al., 2023). Rumination symptoms may be alleviated over the course of antidepressant treatment (Segerberg et al., 2024). However, previous studies have not distinguished between melancholic and anxious subtypes. Therefore, rumination-focused therapies could be tailored differently for melancholic versus anxious subgroups. Our research can contribute to precise and individualized treatment of depression.

Our results demonstrated that rumination in the melancholic subgroup was more serious than in the anxiety subgroup. A tendency for higher CRP and adipokines was observed in atypical depression; increased IL-6 was found in melancholic depression (Křenek et al., 2023). Jessica et al. reported that a posterior cingulate dominant state results in less anticorrelation between the left dorsolateral prefrontal cortex/middle frontal gyrus and the left precuneus/posterior cingulate cortex in melancholic depression is expected to specifically relate to rumination symptoms (Taylor et al., 2022). The at-risk group of depression showed greater activation in two default mode network (DMN) regions, the medial prefrontal cortex and the inferior parietal lobule (IPL), after hearing criticism but not praise. Criticism-specific activation in the IPL was significantly correlated with rumination (Chou et al., 2023). Others confirm the suspected association between rumination and DMN activation, specifically implicating the DMN core regions and the dorsal medial prefrontal cortex subsystem (Zhou et al., 2020). These results are evidence of our research and provide a basis for the neural basis of rumination. However, some but not all studies showed that anxiety symptoms could predict suicide risk (Bjerkeset et al., 2008; Placidi et al., 2000) and worse outcomes with antidepressant treatment (Fava et al., 2008; Uher et al., 2011). Moreover, there were no significant differences among depressive rumination, but patients with bipolar disorder may have had more rumination on positive affect (Kovács et al., 2020).

Negative rumination bias correlates with family history. However, our results are contrary to previous reports. Individuals with depressive symptoms and those with a family history of depression were characterized by higher rumination than controls (Moretta and Messerotti Benvenuti, 2022). This inconsistency is related to the different references. We expect that there are more psychosocial factors in depression without a family history and more biological factors in patients with a family history. Thus, depressive rumination might have more correlations with psychology. Negative memory bias is a depressotypic process and might play a mechanistic role in the development of the co-occurrence of different psychiatric disorders (Duyser et al., 2020). Age of onset correlates with a total score of negative bias, negative memory bias, negative interpretation, and negative rumination bias. Late-life depression was slower to identify surprised faces and more likely to create negative statements in the interpretation task. There was no evidence of negative bias in memory or attention, but patients with late-life depression performed more poorly on the recall task (Baruch et al., 2021). In depression, age of onset was negatively correlated with emotion recognition. It has been suggested that rumination bias is due to underlying negative core beliefs that drive several cognitive processes.

This study provides new evidence of negative bias in depressed patients but did not show a global bias across cognitive domains. These findings may be relevant to patients in primary and secondary care suffering from depression, including inpatients and those presenting a high level of risk. However, we mainly explored melancholic and anxiety subgroups and did not include other subtypes. The exclusion of other depression subtypes (e.g., atypical depression) limits generalizability. Here, we analyzed clinical characteristics associated with the negative cognitive processing bias in patients with MDD. Due to time constraints, we did not conduct long-term follow-up and explore the neural mechanisms of the differences between depressive and anxious rumination. The minimum enrollment criterion is 15 years old. It is possible that the patient could have bipolar disorder later. However, psychiatric diagnosis is dynamic, and it is difficult to define whether it will progress to bipolar disorder. Future studies are needed to explore longitudinal changes in cognitive biases and their treatment responsiveness.

## Data Availability

The raw data supporting the conclusions of this article will be made available by the authors, without undue reservation.
